# High-throughput screening of non-conventional yeasts for conversion of organic waste to microbial oils via carboxylate platform

**DOI:** 10.1038/s41598-024-65150-w

**Published:** 2024-06-20

**Authors:** Mia Žganjar, Mojca Ogrizović, Mojca Matul, Neža Čadež, Nina Gunde-Cimerman, Cristina González-Fernández, Cene Gostinčar, Elia Tomás-Pejó, Uroš Petrovič

**Affiliations:** 1 Department of Molecular and Biomedical Sciences, Jožef Stefan Institute, Ljubljana, Slovenia; 2 Department of Food Science and Technology, Biotechnical Faculty, University of Ljubljana, Ljubljana, Slovenia; 3 Department of Biology, Biotechnical Faculty, University of Ljubljana, Ljubljana, Slovenia; 4 Biotechnological Processes Unit, IMDEA Energy, Madrid, Spain; 5 Institute of Sustainable Processes, Dr. Mergelina, Valladolid, Spain

**Keywords:** Organic wastes, Carboxylates, Oleaginous yeast, Microbial oils, Oleochemicals, Biotechnology, Microbiology

## Abstract

Converting waste into high-value products promotes sustainability by reducing waste and creating new revenue streams. This study investigates the potential of diverse yeasts for microbial oil production by utilizing short-chain fatty acids (SCFAs) that can be produced from organic waste and focuses on identifying strains with the best SCFA utilisation, tolerance and lipid production. A collection of 1434 yeast strains was cultivated with SCFAs as the sole carbon source. Eleven strains emerged as candidates with promising growth rates and high lipid accumulation. Subsequent fermentation experiments in liquid SCFA-rich media, which focused on optimizing lipid accumulation by adjusting the carbon to nitrogen (C/N) ratio, showed an increase in lipid content at a C/N ratio of 200:1, but with a concurrent reduction in biomass. Two strains were characterized by their superior ability to produce lipids compared to the reference strain *Yarrowia lipolytica* CECT124: *Y. lipolytica* EXF-17398 and *Pichia manshurica* EXF-7849. Characterization of these two strains indicated that they exhibit a biotechnologically relevant balance between maximizing lipid yield and maintaining growth at high SCFA concentrations. These results emphasize the potential of using SCFAs as a sustainable feedstock for oleochemical production, offering a dual benefit of waste valorisation and microbial oil production.

## Introduction

The increasing environmental impact of industrial processes, along with efforts to find sustainable and renewable sources for oleochemical production and biofuels have led to the investigation of waste conversion technologies^1–4^. The carboxylate platform is emerging as an important technique for the conversion of organic waste into high-value molecules, particularly with regard to oleochemical production^1,5,6^. Because of concerns about potential disruptions in the food supply chain, caused by the industrial use of edible lipid sources and the extensive use of agricultural land, there has been a shift towards the use of non-edible feedstocks. Waste animal fats, non-edible plant materials and spent cooking oils are increasingly being recognized for their potential in the sustainable production of oleochemicals^2,3,7^. This transition not only mitigates the environmental impact associated with waste but also promotes the development of high-value molecules, creating new opportunities to generate revenue without depleting food resources^6,8^.

Among the numerous biological resources, microbial oils, also known as single-cell oils, stand out as a versatile and potentially sustainable option. While their application has traditionally been considered for the energy sectors^2,3,5,8^, the broader potential of microbial oils extends far beyond this, including their use in the food industry, pharmaceuticals, and as precursors for a variety of oleochemicals, such as polymers and lubricants^9–11^.

The use of oleaginous microorganisms, either bacteria, fungi, or algae, has become a focus of research. Among oleaginous microorganisms, yeasts are often listed as the most promising source of microbial oils for the production of oleochemicals^2,3,5,7^. Traditionally, yeasts were considered oleaginous if their intracellular lipid content reached or exceeded 20% of their cell dry weight (CDW)^12–14^. However, based on new evidence, a new definition has been proposed, based on which the term can be expended to yeasts whose intracellular or extracellular lipids are above the arbitrary 20% threshold in at least one of the cultivation conditions and should be applied to individual strains rather than to whole species^13^.

While oleaginous yeasts are known for their ability to convert simple sugars into high quantities of intracellular lipids under nitrogen-, phosphorus- or iron-limiting conditions, the use of simple sugars as the main substrate leads to high costs, rendering the microbial oil production economically unfeasible^12,14,15^. Alternative, low-cost substrates have been proposed, including but not limited to the use of volatile organic fatty acids with short-chain aliphatic tails of 2–6 carbon atoms (C2-C6), also called short-chain fatty acids (SCFAs). These SCFAs can be obtained from anaerobically fermented bio-waste such as food waste, sludge, and waste waters^2,8,12,13,16–20^. An economically viable process of microbial oil production could therefore entail a conversion of SCFAs into microbial oils in the form of intracellular storage lipids from oleaginous yeasts. There are currently 160 known yeast species^15^ with at least one representative oleaginous strain. Most biotechnologically important and industrially utilized are specific strains of *Yarrowia lipolytica* and *Rhodotorula toruloides*, although other species have gained much attention in recent years^12–14^. In comparison, only a few yeast species have been identified for their ability to utilize SCFAs: *Y. lipolytica, Cutaneotrichosporon oleaginosum, Cutaneotrichosporon curvatum, Naganishia albida, Trichosporon cutaneum, Saccharomyces cerevisiae, Rhodotorula toruloides, Rhodotorula glutinis, Apiotrichum brassicae* and *Pichia kudriavzevii*^2,12,13,16–21^. In particular, strains such as *Y. lipolytica* ACA DC 50 109, *Y. lipolytica* CICC 31,596, *C. curvatum* NRRL Y-1511, and *C. curvatum* ATCC 20,509 have a considerable ability to utilize SCFAs and accumulate lipids^22–24^. The strains *Y. lipolytica* ACA DC 50 109 and *C. curvatum* NRRL Y-1511 have been shown to accumulate up to 78% w/w and 57% w/w lipids, respectively, when cultivated on SCFAs at concentrations of up to 19 g/L and optimised carbon to nitrogen (C/N) ratio^24^. Furthermore, strain *C. curvatum* ATCC 20,509 has been reported to accumulate 65% w/w lipids when cultivated with 40 g/L of acetic acid and up to 57% w/w when cultivated on a mixture of SCFAs at a concentration of 30 g/L^22,25,26^. The strain *Y. lipolytica* CICC 31,596 can accumulate 30% w/w lipids in a mixture of SCFAs with a total concentration of up to 50 g/L and adjusted alkaline pH^22,23^.

Recent research has therefore shown substantial progress in optimizing the use of SCFAs for microbial oil production. The key factors influencing this process are high SCFAs concentrations and their toxicity, as well as process variables such as carbon to nitrogen (C/N) ratio, pH, temperature, oxygen availability, as well as nitrogen source. The optimization of these factors as well as managing high and varying SCFA concentrations in waste streams and the specific SCFA profiles of different yeast strains highlight the need for further development and possibly strain-specific optimisation. In addition, genetic variation among strains of the same species has a significant impact on SCFA utilization and lipid production. This emphasizes the importance of strain selection and genetic engineering for the improvement of oleochemical synthesis^22^.

While several studies have explored the utilization of SCFAs, no systematic and extensive survey of phylogenetically diverse yeast taxa that can utilize SCFAs has been conducted to date. We aimed to address this issue by screening a collection of 1434 yeast strains for their ability to utilize SCFAs at high concentrations and different ratios of individual SCFAs, to expand the understanding of yeast metabolic diversity. To further advance oleochemical synthesis, we aimed to find a suitable oleaginous strain that could efficiently convert SCFAs into microbial oils. The term ‘yeasts’ is used in this article also for yeast-like fungi of Pezizomycotina which can grow in the form of single cells at least under certain conditions.

## Results and discussion

### Initial assessment of growth on SCFAs

**Figure 1:**
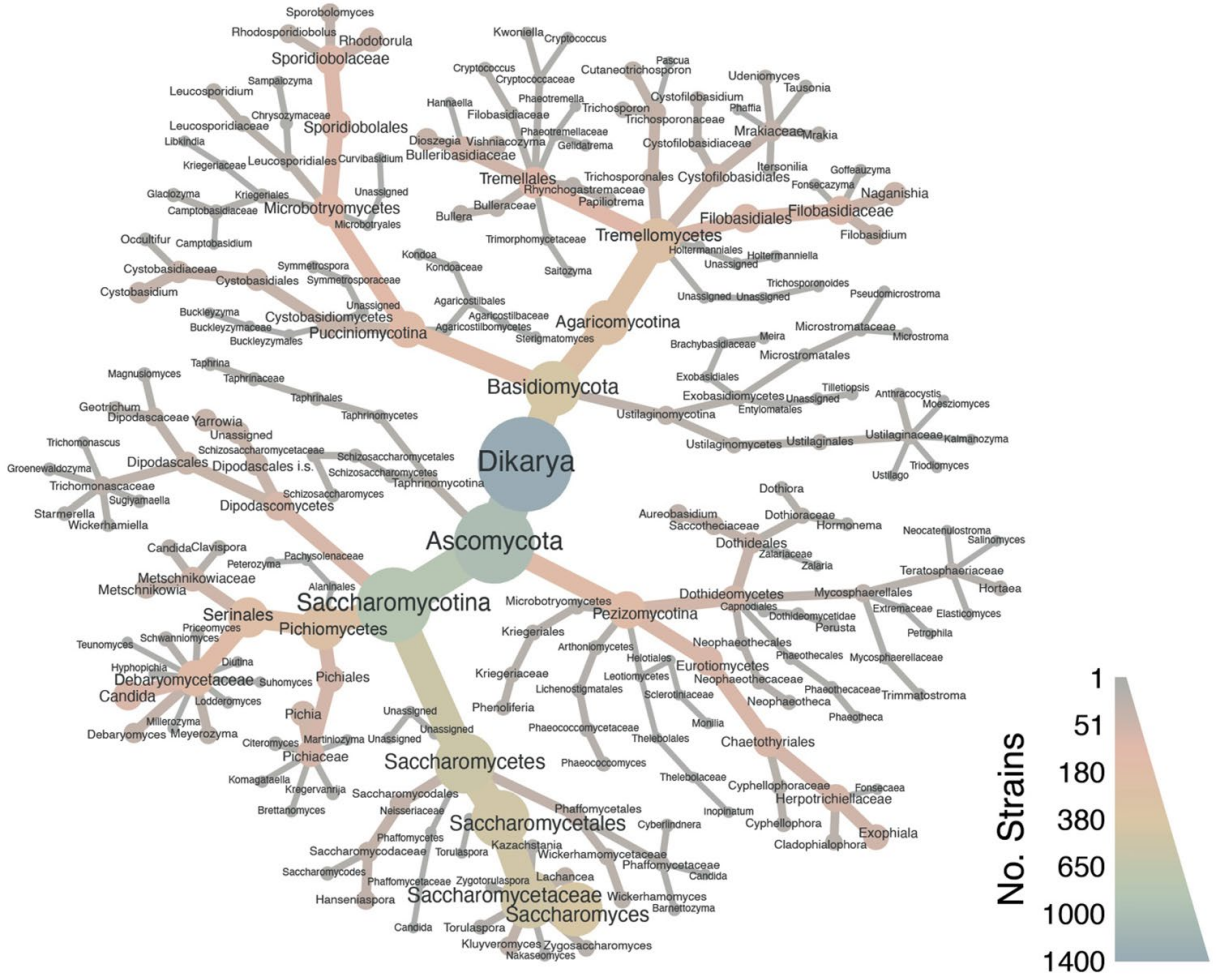
Taxonomic diversity of 1434 yeast strains at genus level tested for their ability to grow on SCFAs. Colour scale and node size denote number of strains per taxa used in the study. Spatial arrangement of taxonomic groups does not imply phylogenetic distances.

In the first screening, a total of 1434 diverse natural yeast strains (Fig. 1, Supplementary S2 Figures S1 and S2) were subjected to a high-throughput growth assay on media containing three different mixtures of acetic acid (A), propionic acid (P) and butyric acid (B) at a total SCFA concentration of either 15 g/L or 25 g/L. Medium with glucose as the sole source of carbon was used as control. The choice of different mixtures of SCFAs in varying ratios was driven by typical waste stream compositions^22,23^, and by the objective to investigate the effects of varying chain lengths and ratios on yeast growth and lipid synthesis.

**Figure 2:**
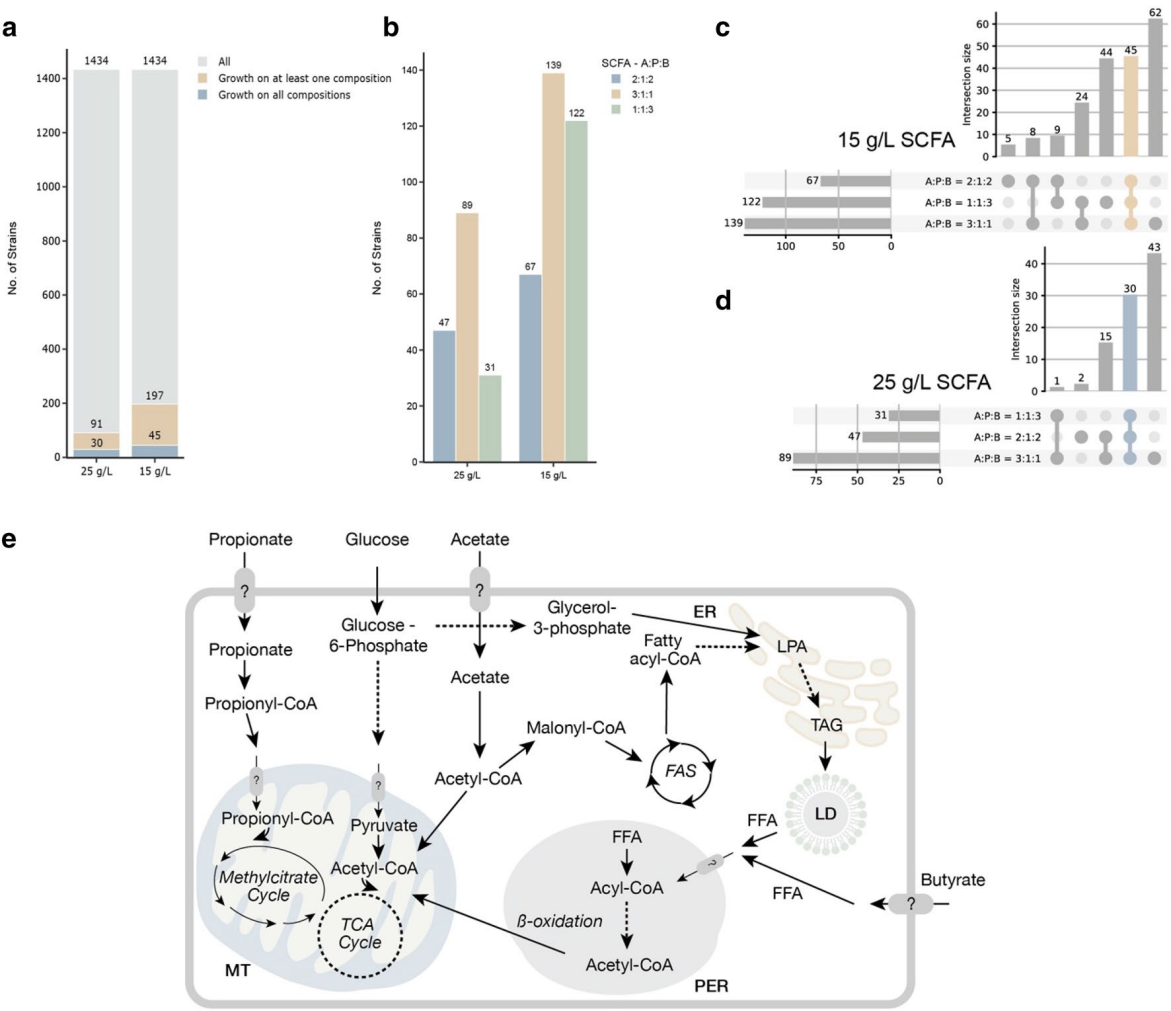
Initial evaluation of yeast growth on SCFA-rich media. (**a**) Number of yeast strains capable of growth on at least one or all of the three media compositions per total SCFA concentration (15 g/L and 25 g/L). (**b**) Distribution of growth across various SCFA media compositions. Three mixtures were tested: A:P:B = 2:1:2, A:P:B = 3:1:1 and A:P:B = 1:1:3, corresponding to the ratios between individual SCFAs: acetic acid (A), propionic acid (P) and butyric acid (B) respectively. (**c**) and (**d**) UpSet plots of intersectional representations of yeast strains capable of growing on tested SCFA media compositions by total SCFA concentration at 15 g/L and 25 g/L respectively. Lines connecting the dots represent the overlap between media compositions, and the intersection size indicates the number of strains capable of growing on one, two, or all three compositions. (**e**) Schematic diagram of SCFA utilisation. The solid and dashed arrows signify single and multiple steps, respectively. The following abbreviations were used: *LPA* lysophosphatidic acid, *TAG* triacylglycerol, *LD* lipid droplet, *FFA* free fatty acid, *FAS* fatty acid synthase, *TCA* Cycle tricarboxylic acid cycle, *MT* mitochondrion, *ER* endoplasmic reticulum, *PER* peroxisome. The outer cell membrane is indicated in grey.

Of 1434 strains, the first screening identified only 91 (6.35%) capable of growth on at least one media with a total SCFA concentration of 25 g/L (Fig. 2a). Of these, 30 (2.1%) strains grew on all three media compositions tested (Fig. 2a, d). A larger number of strains, 197 (13.74%) grew on at least one medium with SCFA concentration of 15 g/L, with 45 strains (3.14%) growing on all three media compositions (Fig. 2a, c).

The largest number of strains grew on the media with A:P:B = 3:1:1 with a higher proportion of acetic acid at both total SCFA concentrations tested (Fig. 2b–d). This suggests that acetic acid may be a favourable carbon source for most tested yeast strains, highlighting the influence of the SCFA distribution profile on yeast proliferation. These results agree with previous reports showing that the preference for acetic acid in *Y. lipolytica*^17,20,27^, *Naganishia albida (Cryptococcus albidus)*^28,29^ and *Cutaneotrichosporon curvatum (Cryptococcus curvatus*)^25,26,30^. In contrast, growth results for the other two media compositions, A:P:B = 2:1:2 and A:P:B = 1:1:3, showed no clear patterns. The overlaps of yeast strain growth across tested SCFA media compositions are presented in Fig. 2c and d. Interestingly, all but one of the strains (30 strains) that were able to grow on the composition with a higher proportion of butyric acid (A:P:B = 1:1:3) at the highest of the two SCFA concentrations tested (25 g/L), were also able to grow on other two compositions. These results suggest that the A:P:B = 1:1:3 composition at a total SCFA concentration of 25 g/L may be the most stringent testing condition with toxicity and growth arrest at higher concentrations of butyric acid, which has also been reported in previous studies^20,30^. However, there were 44 strains out of 122 that were capable of growing only on the A:P:B = 1:1:3 composition, but at a lower total SCFA concentration (15 g/L) (Fig. 2c). Tolerance to different SCFAs could therefore be species- or even strain-specific. However, it was previously shown that acetic and butyric acids can increase the biomass of *Y. lipolytica* and *C. curvatum*, compared to propionic acid, but only below the toxicity level of butyric acid and at appropriate ratios^27,31^.

Following the high-throughput screening with a robotic pinning replicator, 571 yeast strains with inconsistent growth were manually inoculated. These were mostly yeast strains that formed pseudohyphae or exhibited filamentous growth on the selected media. To ensure the reliability of the observed growth, this subset also included 35 strains of the genus *Yarrowia*, which were subjected to growth tests on all three SCFA media compositions at the established total concentration of 25 g/L. Of the 35 *Yarrowia* strains, 30 strains showed growth within 3–5 days. Among these, 19 strains exhibited the ability to grow across all three media compositions and 27 *Yarrowia* strains were able to grow solely on two compositions—on A:P:B = 2:1:2 and A:P:B = 3:1:1. One strain was able to grow only on the composition A:P:B = 3:1:1 and 2 strains only on the A:P:B = 2:1:2. Other 536 non-*Yarrowia* strains were tested solely on the media composition A:P:B = 1:1:3 at a SCFA concentration of 25 g/L, previously identified as the most stringent condition. Only 20 strains presented a delayed biomass formation after a 3-week incubation period, making them not only slow growers, but also incomparable with the reference strain *Y. lipolytica* CECT1240. Therefore, these strains were excluded from further analyses. For the majority of manually tested strains (521 strains), no growth was observed even after an incubation period of 4 weeks.

### Quantitative growth assessment and lipid profiling in the second screening round

After the first large-scale screening, the strains were roughly grouped according to their broad growth range, guiding the selection of best-performing strains. Based on the initial screening, the 54 most promising candidate strains were selected for subsequent rounds of growth and neutral lipid accumulation tests.

**Figure 3:**
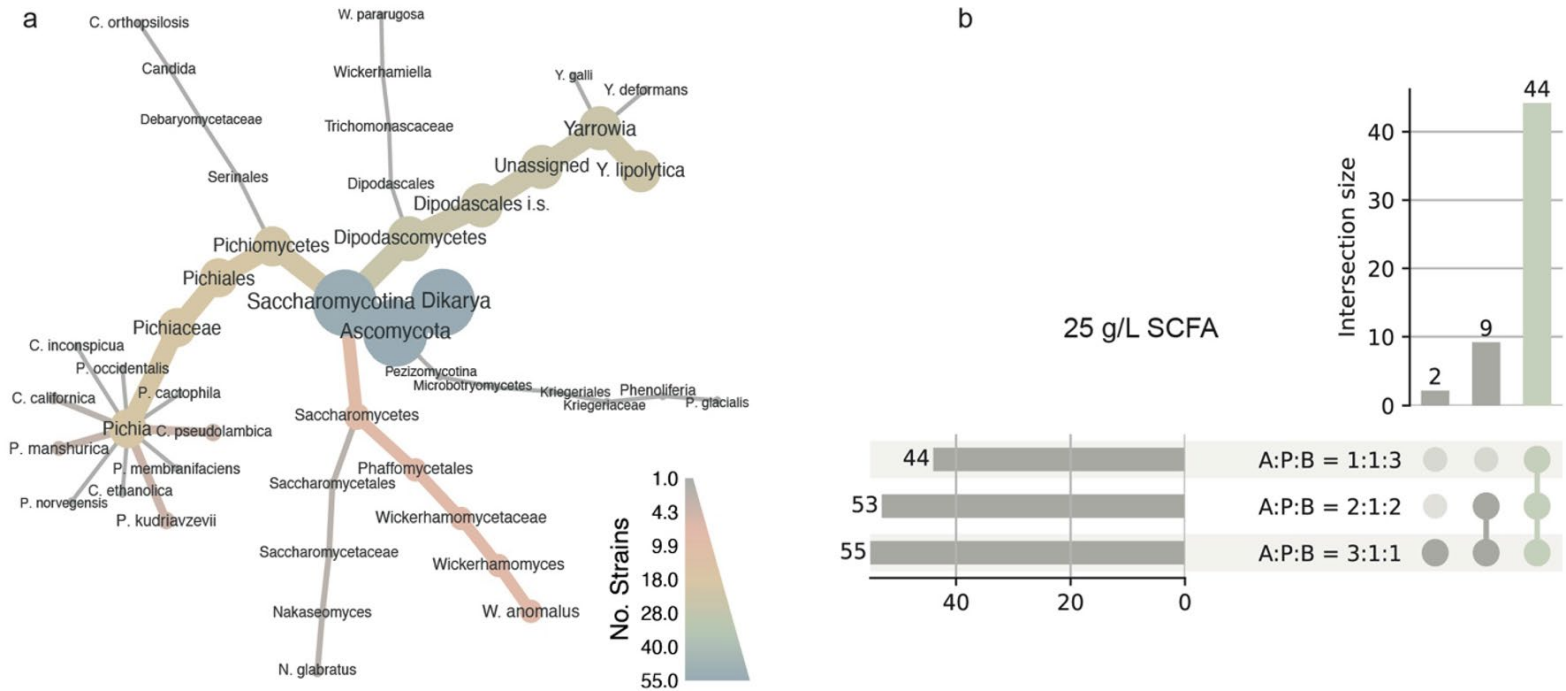
Taxonomic distribution and growth evaluation of selected candidate yeast strains. (**a**) Taxonomic diversity of best-performing candidate species from the initial large-scale screening, including the reference *Y. lipolytica* CECT1240 strain, illustrating the diversity of the candidate species. Colour scale and node size denote number of strains per taxa. (**b**) Intersectional representation of growth patterns among the candidate strains and the reference strain (54 + 1) in the second screening round. The lines connecting the dots represent the overlap between media compositions, and the intersection size indicates the number of strains that grow either on one, two or all three of SCFA media compositions: acetic acid (A) : propionic acid (P) : butyric acid (B) = 2:1:2, A:P:B = 3:1:1 and A:P:B = 1:1:3, at final concentration of 25 g/L.

The taxonomic distribution of the selected strains was limited to seven genera (Fig. 3a). Not counting the reference strain *Y. lipolytica* CECT1240, the taxonomic genera with the most yeast strains, were *Yarrowia* (23) and *Pichia* (19). The genus *Wickerhamomyces* was represented by seven strains (all *W. anomalus*) and the genus *Nakaseomyces* by two (both *N. glabratus*). The genera *Candida*, *Phenoliferia*, and *Wickerhamiella* were represented by a single strain per genus (Supplementary S2 Table S1). Among the already known oleaginous yeast species, our set of candidate strains contained three species of the genus *Yarrowia (Y. lipolytica, Y. deformans, Y. galli*), five species of the *Pichia* clade (*P. kudriavzevii, P. cactophila, P. manshurica, P. occidentalis, [Candida] pseudolambica)*, one *Candida* species (*C. orthopsilosis,*) and *Phenoliferia glacialis (Rhodotorula glacialis)*^12–14^ (Supplementary S2 Table S1).

In the second screening, we quantitatively assessed the growth on SCFAs on the above described candidate strains in more detail. Fitness values (endpoint colony sizes), lag phase duration (lag variable) and time point at maximal growth rate (t_max variable) were computed for each strain on each of the three aforementioned SCFA-rich media compositions with a concentration of 25 g/L.

The growth pattern of the strains that were subjected to the second screening is presented in Fig. 3b. Of the 54 candidate yeast strains, 43 (79.6%) strains were able to grow on all three compositions, 9 (16.7%) strains were able to grow on the compositions A:P:B = 2:1:2 and A:P:B = 3:1:1, and 2 (3.7%) strains were able to grow exclusively on the A:P:B = 3:1:1 composition. Similar to the initial screening, acetic acid proved to be the most favourable SCFA tested.

We then compared the fitness values of strains grown on SCFA-rich media compositions relative to their growth on glucose media, designated as relative fitness. The median relative fitness values of strains growing on A:P:B = 3:1:1, A:P:B = 1:1:3 and A:P:B = 2:1:2 compositions compared to growth on glucose were 26.9%, 26.5% and 19.3%, respectively. The median fitness values in the pixel intensities of the compositions A:P:B = 3:1:1, A:P:B = 1:1:3 and A:P:B = 2:1:2 were 1549 (n = 55), 1458 (n = 44) and 1102 (n = 53), respectively (Supplementary S2 Figure S3 a). Comparing fitness distributions per medium showed minimal differences between the compositions, although 11 strains were not able to grow on the composition A:P:B = 1:1:3 and 2 on the composition A:P:B = 2:1:2. More prominent differences were detected in the time point of maximum growth (t_max), where maximum growth on the composition A:P:B = 3:1:1 occurred on average after 47 h, in contrast to 74 h on the composition A:P:B = 2:1:2 and 82 h on the composition A:P:B = 1:1:3 (Supplementary Figure S3 c). The distributions of t_max indicate a grouping of the strains into fast and slow growing groups, which is also consistent with the distribution of lag phase duration (Supplementary Figure S3 b). For propionic and butyric acid, lower utilisation rates and longer lag phase compared to acetic acid were previously reported^27^. These were attributed to different metabolic routes after uptake^27,32^. On the one hand, acetic acid is directly converted to acetyl-CoA, which either enters the de novo lipid synthesis pathway, where it is used to produce fatty acids and subsequently triacylglycerols, or it is channelled into the mitochondria for energy production (Fig. 2e). Propionic acid, on the other hand, requires additional conversion steps via the methyl citrate cycle to form acetyl-CoA. Upon entrance into the cell, butyric acid is initially converted to butyryl-CoA and subsequently transformed into acetyl-CoA via the β-oxidation pathway, which can contribute to both de novo and ex novo lipid synthesis. In de novo synthesis, acetyl-CoA produced from SCFAs is integrated into the fatty acid biosynthetic pathway under nutrient-limited conditions, whereas in *ex novo* synthesis, fatty acids are directly incorporated into lipids. These pathways illustrate the theoretically different efficiencies of SCFAs^19,33,34^.

Similarly to distributions of t_max, the strains could also be roughly divided into two groups according to their accumulation of neutral lipids, with a high and a low lipid accumulation capacity with an average neutral lipid accumulation of 4211 RFUs (Supplementary S2 Figure S3 d).

**Figure 4:**
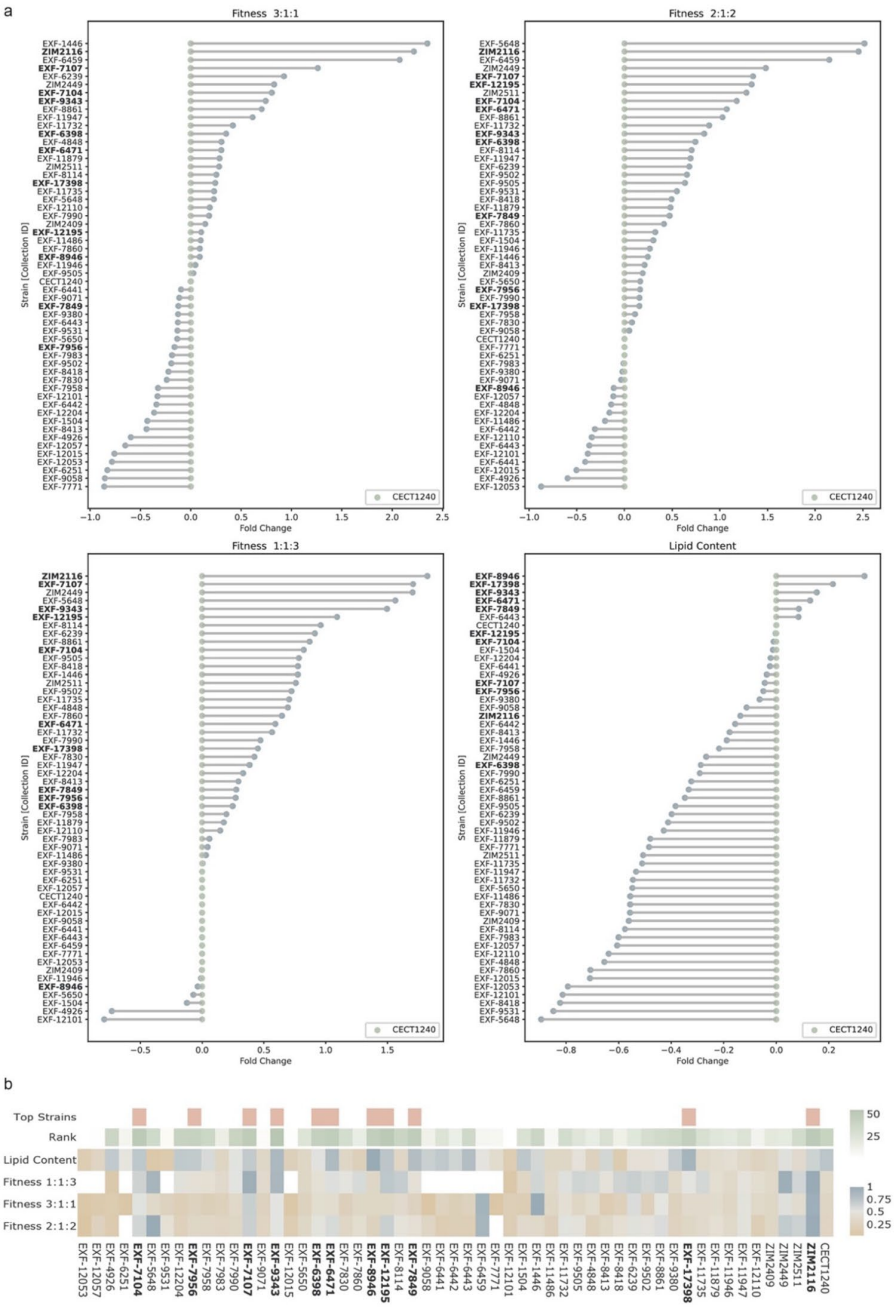
Comparison of fitness and lipid content of the candidate strains to the reference *Y. lipolytica* CECT1240 strain. (**a**) Fold change in fitness across different tested SCFA media compositions (A:P:B = 3:1:1, 2:1:2, and 1:1:3) and lipid content depicted for selected yeast strains (blue dots). Media compositions present the ratio between individual SCFAs: acetic (A), propionic (P) and butyric acid (B), in this order. The bars represent fold changes relative to the CECT1240 strain, denoted with green dots. (**b**) Heat-map od weighted ranks, as well as scaled fitness and lipid accumulation data. Weighted ranks are based on a combined score, considering both traits, giving lipid accumulation greater importance (fitness:lipids = 40:60%). The colour gradient represents the rank of each strain, with darker colours indicating higher ranks. Top strains with the best overall performance are denoted in red.

To obtain the final subset of candidate strains and effectively compare them with the reference strain, the growth parameters, as well as the neutral lipid accumulation RFUs were normalized to the reference strain *Y. lipolytica* CECT1240 and fold change values were calculated. Most candidate strains exhibited a higher fitness compared to the reference strain, with several strains showing a fold change exceeding 1.5, suggesting an improved growth rate on the tested SCFA-rich media compositions (Fig. 4a). In particular, *Y. lipolytica* strain ZIM2116 showed a high fitness on all compositions.

We further inspected the relationship between fitness and the time at maximum growth (t_max). This relationship might provide additional insight into the strains that have a growth advantage over the reference strain in certain media compositions (Supplementary S2 Figure S4 a–c). Strains with higher fitness tend to reach maximum growth faster than less fit strains, suggesting that a faster time to reach maximum growth rate is associated with better fitness^35^. The fittest strains therefore have greater fitness and smaller t_max fold changes compared to the reference strain. A more prominent deviation between fast growing and slow growing strains can be observed within the compositions A:P:B = 3:1:1 and A:P:B = 2:1:2. A subset of strains is clustered below and to the left of the reference strain, indicating that they not only surpass the reference strain in fitness, but also reach maximum growth rate sooner. In the composition A:P:B = 1:1:3, however, the dots are more scattered, indicating a consistent pattern of increased fitness and reduced t_max relative to the reference strain (Supplementary S2 Figure S4 c). A clustering of strains around zero indicates a proportion of strains that did not deviate substantially from the reference strain.

Further, we examined the fold change in neutral lipid content of the candidate strains compared to the reference strain (Fig. 4a). In this case, only six strains accumulated a higher proportion of neutral lipid compared to the reference strain, exceeding it by 8% to 34%. The results demonstrate that, although many candidate strains outperformed the reference strain in relation to their growth and utilisation of SCFAs, their ability to accumulate neutral lipids to a high extent is relatively limited.

For the final selection step, neutral lipid accumulation was prioritized over SCFA utilisation, while growth-related parameters were still considered. We subjected the phenotype data to a scaling and a weighted ranking procedure, in which lipid accumulation was given a 60% weight and remaining 40% weight to fitness and t_max growth parameters (Fig. 4b).

A sub-selection of the 11 best strains with significantly higher fitness compared to the reference strain is presented in Table 1. This is emphasized both by the magnitude of fold changes and the statistical tests. It should be noted that although not all strains reached statistical significance (Supplementary S1) with the BH-adjusted *p* value < 0.05 across fitness and lipid content, potential phenotypic variation of the strains might be explored further.

**Table 1:** Subset of candidate strains with significant fitness differences comparing to the reference Y. lipolytica CECT1240 strain.

Strain ID	Species	Parameter	SCFA Composition	Fold Change	U-stat	*p* value	p.adj_BH	Rank
EXF-9343	*Pichia kudriavzevii*	Fitness	A:P:B = 3:1:1	0.74	1.00	2.58e-04	3.98e-03	55
EXF-7104	*Nakaseomyces glabratus*	Fitness	A:P:B = 3:1:1	0.80	0.00	1.29e-04	2.32e-03	53
ZIM2116	*Yarrowia lipolytica*	Fitness	A:P:B = 2:1:2	2.45	0.00	4.00e-04	4.12e-02	52
ZIM2116	*Yarrowia lipolytica*	Fitness	A:P:B = 3:1:1	2.22	15.00	1.93e-03	2.09e-02	
EXF-7107	*Pichia kudriavzevii*	Fitness	A:P:B = 3:1:1	1.26	0.00	1.29e-04	2.32e-03	51
EXF-6471	*Pichia kudriavzevii*	Fitness	A:P:B = 3:1:1	0.30	6.00	3.74e-03	2.69e-02	49
EXF-17398	*Yarrowia lipolytica*	Fitness	A:P:B = 3:1:1	0.24	3.00	9.03e-04	1.30e-02	47
EXF-6398	*Pichia kudriavzevii*	Fitness	A:P:B = 3:1:1	0.35	5.00	2.45e-03	2.12e-02	46

The table lists selected yeast strains with their corresponding Collection ID and Scientific Name. The fitness fold change, relative to the reference strain is reported along with the Mann–Whitney U statistic (U-stat) and the associated *p* value. *p* values were adjusted for multiple comparisons using the Benjamini-Hochberg (p.adj_BH) correction. The final column presents the weighted rank of each strain, ranging from 1 to 55, where rank 55 denotes the highest overall performance.

With this approach the initial set of over 1,400 strains was reduced to the top 11 candidates for further fermentation experiments in SCFA-rich liquid media.

### Yeast growth and microbial oils production in liquid SCFA-rich media

Although previous studies have suggested that mixtures of SCFAs, containing acetic, propionic, and butyric acids, can significantly impair yeast growth at concentrations of 5 g/L^18^, and can even completely inhibit yeast growth when the concentrations of propionic and butyric acids concentrations reach 10 g/L and 5 g/L, respectively^20^, it has recently been reported that *Y. lipolytica* is able to grow in a mixture of SCFAs at a concentration of 15 g/L^17,36,37^.

On this basis, the selected and reference strains were first grown in the presence of 15 g SCFAs/L in a 3:1:1 ratio of acetic, propionic, and butyric acids according to the acetic acid preference demonstrated in the high-throughput screening.

**Figure 5:**
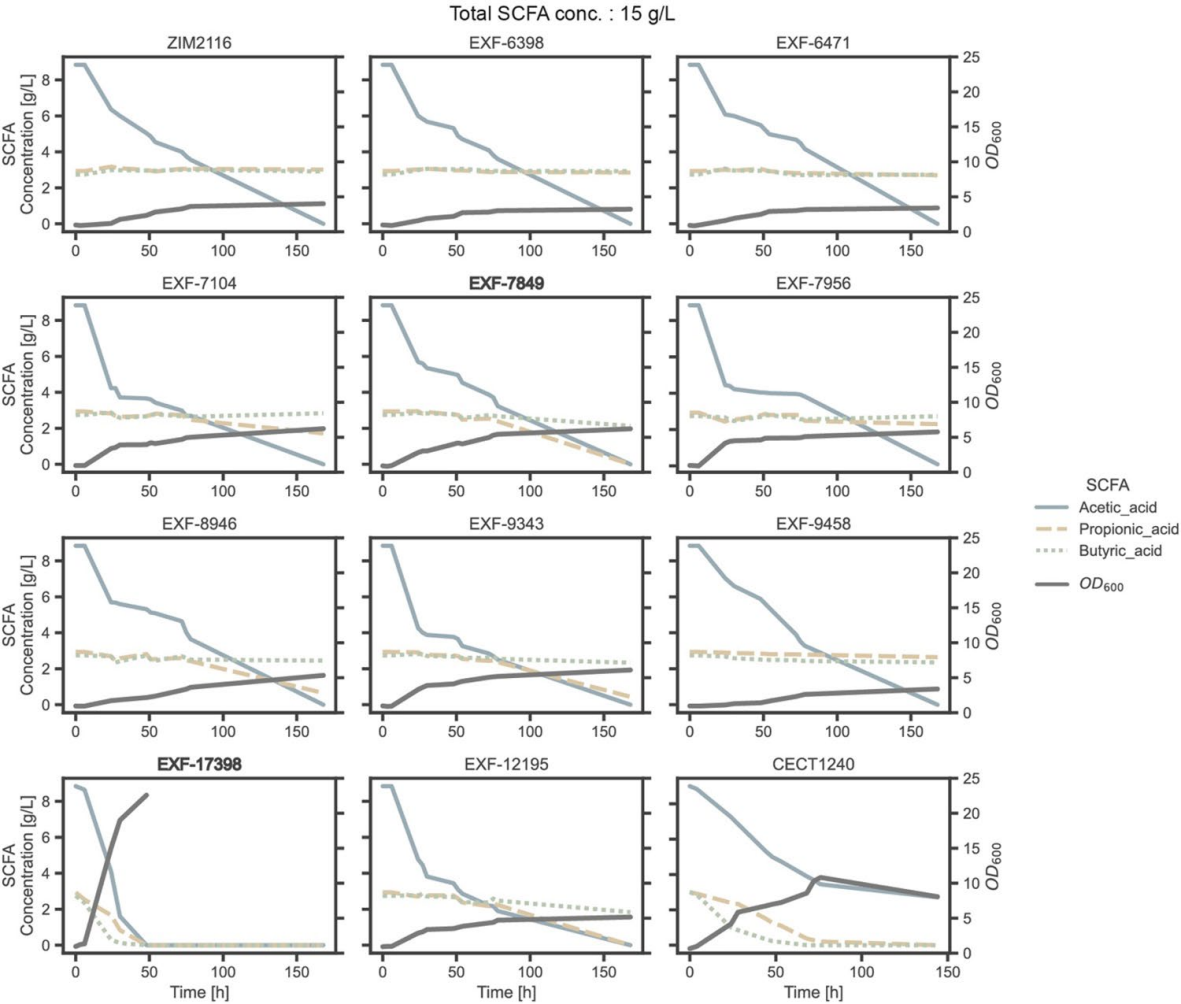
Consumption of short-chain fatty acids (SCFAs) and growth comparison in media composition A:P:B = 3:1:1 containing 15 g/L SCFAs. Presented fermentation results of the most promising 11 strains from the screening process with the reference Y. lipolytica CECT1240 strain. Acetic, propionic and butyric acid consumptions and optical density at 600 nm (OD_600_) are shown through time. The two most promising strains are highlighted with bold subtitles.

All the strains were able to grow in media containing 15 g/L SCFAs (Table 2). Acetic acid was completely metabolized by the 11 strains, albeit at different consumption rates (Fig. 5). In addition, *P. manshurica* EXF-7849, *Y. lipolytica* EXF-17398 and the reference strain completely consumed all available propionic acid, while *P. norvegensis* EXF-12195, *P. kudriavzevii* EXF-9343, and *P. membranifaciens* EXF-8946 consumed 85%, 85% and 78%, respectively. Butyric acid was consumed completely only by the *Y. lipolytica* EXF-17398 and the reference strain, while the other strains consumed less than 10%, with the exception of *P. manshurica* EXF-7849 and *P. norvegensis* EXF-12195, which consumed 23% and 33% of butyric acid, respectively.

**Table 2:** Short-chain fatty acids (SCFAs) consumption, maximum optical density (OD_600_), and lipid content of the 11 most promising yeast strains, analysed at the end of cultivation in the screening media containing a total of 15 g/L SCFAs and A:P:B = 3:1:1.

Strain ID	Species	SCFAs (%)				Maximal OD_600_	Lipid (% w/w)
		Acetic acid	Propionic acid	Butyric acid	Total		
CECT1240*	*Yarrowia lipolytica*	70	100	100	82	10.8 ± 0.4	15.9 ± 1.0
ZIM-2116	*Yarrowia lipolytica*	100	< 10	< 10	60	4.0 ± 0.1	15.4 ± 0.2
EXF-6398	*Pichia kudriavzevii*	100	< 10	< 10	60	3.2 ± 0.1	15.9 ± 0.2
EXF-6471	*Pichia kudriavzevii*	100	8	< 10	63	3.0 ± 0.1	18.4 ± 0.2
EXF-7104	*Nakaseomyces glabratus*	100	41	< 10	69	6.2 ± 0.1	18.2 ± 0.2
EXF-7849	***Pichia manshurica***	**100**	**100**	**23**	**85**	**6.2 ± 0.1**	**19.9** ± **0.2**
EXF-7956	*Pichia [Candida] pseudolambica*	100	22	< 10	65	5.8 ± 0.1	14.5 ± 0.2
EXF-8946	*Pichia membranifaciens*	100	78	11	79	5.3 ± 0.1	15.7 ± 0.2
EXF-9343	*Pichia kudriavzevii*	100	85	15	81	6.1 ± 0.1	15.7 ± 0.2
EXF-9458	*Pichia kudriavzevii*	100	10	14	66	3.4 ± 0.1	18.2 ± 0.2
EXF-17398	***Yarrowia lipolytica***	**100**	**100**	**100**	**100**	**22.6 ± 0.2**	**24.7** ± **0.2**
EXF-12195	*Pichia norvegensis*	100	85	33	81	5.2 ± 0.1	16.7 ± 0.2

The reference strain is indicated with *.

The two most promising strains are in bold.

In terms of growth, maximum optical density of *Y. lipolytica* EXF-17398 reached higher values than the reference strain CECT1240 (OD_600_ of 24.7 compared to OD_600_ of 15.9). The other strains showed maximum OD_600_ values between 3.0 and 6.2.

Regarding the accumulation of intracellular lipids, *Y. lipolytica* EXF-17398 and the reference strain accumulated lipids to 24.7 ± 0.2% w/w and 15.9 ± 1.0% w/w, respectively (Table 2, Fig. 7). The other strains were able to accumulate between 14.5% and 18.4%, except for *P. manshurica* EXF-7849, which achieved an accumulation of almost 20% of lipids.

Remarkably, all strains were able to grow in the liquid medium containing 15 g/L of SCFAs, indicating a high tolerance of the tested strains to SCFAs. Arslan et al. showed that increasing the SCFAs concentration from 10 to 15 g/L improved yeast growth and had no significant effect on lipid production^37^. Llamas et al. showed that increasing the SCFAs concentration up to 26.5 g/L, enabled the growth of yeasts, but such a high concentration reduced the growth by 45%^19^. Therefore, to analyse the potential inhibitory or beneficial effects of increasing the SCFAs concentration, a new experiment was performed using a synthetic medium containing 25 g/L SCFAs with the same profile of acetic, propionic, and butyric acids (A:P:B = 3:1:1).

**Figure 6:**
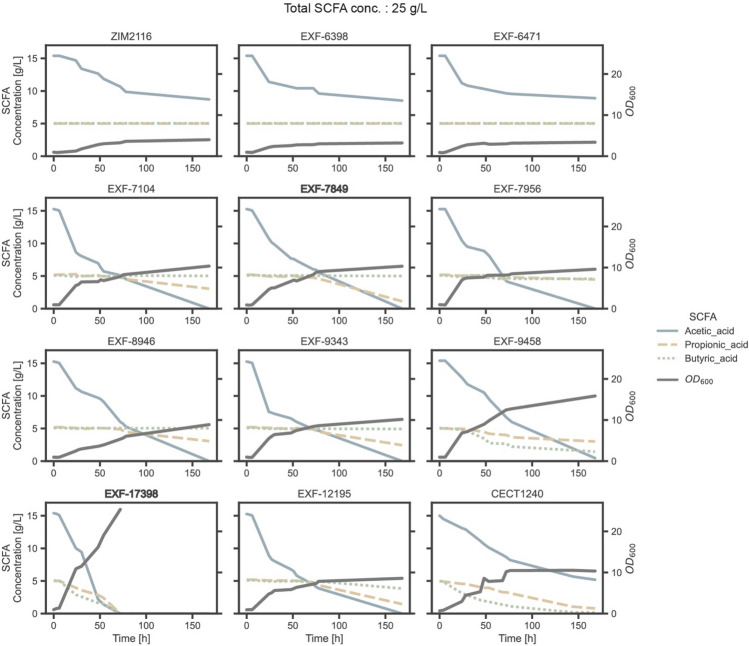
Consumption of short-chain fatty acids (SCFAs) and growth comparison in media composition A:P:B = 3:1:1 containing 25 g/L SCFAs. Presented fermentation results of top 11 strains from the screening process with the reference CECT1240 strain. Acetic, propionic and butyric acid consumptions and optical density at 600 nm (OD_600_) shown through time are shown. The two most promising strains are highlighted with bold subtitles.

All the strains were able to grow in the media containing 25 g/L SCFAs (Fig. 6, Table 3). However, in contrast to the previous experiment with 15 g/L of SCFAs, not all strains were able to metabolize all available acetic acid. In particular, strains *Y. lipolytica* ZIM2116, *P. kudriavzevii* EXF-6398 and *P. kudriavzevii* EXF-6471 only metabolized between 43 and 45% of the available acetic acid. Similar to the previous experiment, only *Y. lipolytica* EXF-17398 was able to completely consume propionic acid. Notably, the reference strain *Y. lipolytica* CECT1240, *Y. lipolytica* EXF-7849 and *P. norvegensis* EXF-12195 strains consumed 84, 78 and 72%, respectively. In contrast, strains *Y. lipolytica* ZIM2116, *P. kudriavzevii* EXF-6398 and *P. kudriavzevii* EXF-6471 showed a propionic acid consumption of less than 10%. The remaining five strains consumed between 40 and 53% of propionic acid.

**Table 3:** Consumption of short-chain fatty acids (SCFAs), maximum optical density (OD_600_), and lipid content of the most promising 11 yeast strains, analysed at the end of cultivation in the screening media containing a total of 25 g/L SCFAs and A:P:B = 3:1:1.

Collection ID	Scientific name	SCFAs (%)				Maximal OD_600_	Lipid (% w/w)
		Acetic acid	Propionic acid	Butyric acid	Total		
CECT1240*	*Yarrowia lipolytica*	65	84	97	75	10.5 ± 0.3	15.0 ± 0.5
ZIM-2116	*Yarrowia lipolytica*	43	< 10	< 10	26	4.0 ± 0.1	12.8 ± 0.2
EXF-6398	*Pichia kudriavzevii*	45	< 10	< 10	27	3.2 ± 0.1	14.6 ± 0.2
EXF-6471	*Pichia kudriavzevii*	43	< 10	< 10	26	3.4 ± 0.1	16.6 ± 0.2
EXF-7104	*Nakaseomyces glabratus*	100	41	< 10	65	10.4 ± 0.1	16.9 ± 0.2
EXF-7849	***Pichia manshurica***	**100**	**78**	** < 10**	**76**	**10.3 ± 0.1**	**16.8 ± 0.2**
EXF-7956	*Candida pseudolambica*	100	41	< 10	65	9.6 ± 0.1	11.6 ± 0.2
EXF-8946	*Pichia membranifaciens*	100	41	< 10	65	8.9 ± 0.0	13.9 ± 0.1
EXF-9343	*Pichia kudriavzevii*	100	53	< 10	71	10.2 ± 0.1	12.9 ± 0.1
EXF-9458	*Pichia kudriavzevii*	97	40	71	81	15.9 ± 0.1	17.4 ± 0.2
EXF-17398	***Yarrowia lipolytica***	**100**	**100**	**100**	**100**	**25.4 ± 0.3**	**26.5 ± 0.2**
EXF-12195	*Pichia norvegensis*	100	72	25	80	10.4 ± 0.1	15.1 ± 0.1

The reference strain is indicated with *.

The two most promising strains are in bold.

Butyric acid was completely consumed by *Y. lipolytica* EXF-17398 and almost completely by the reference strain *Y. lipolytica* CECT1240 (100% and 97%), while *P. kudriavzevii* EXF-9458 also showed a high consumption of 71%. The remaining eight strains showed a consumption of less than 10%, highlighting the difficulty of metabolizing butyric acid in this mixture. This consistent with the results of previous studies, in which Gao et al*.*^20^ observed that the order of toxicity of SCFAs was butyric acid > propionic acid > acetic acid.

In another recent study of *Morales-Palomo* et al.^17^, which investigated the metabolic capabilities of the strain *Y. lipolytica* ACA DC 50,109 in both synthetic and anaerobic digestion-derived media containing SCFAs, a preference for the consumption of acetic acid over butyric or hexanoic acid was also reported. When examining the consumption rate of a mixture of SCFAs containing acetic, butyric, and caproic acid by *Y. lipolytica* W29, it was observed that the highest consumption rate was associated with acetic acid^38^. Conversely, the consumption of butyric and caproic acids was significantly lower in comparison, especially in the case of caproic acid. This suggests that the metabolism of these oleaginous yeasts is optimized to preferentially metabolize short-chain SCFAs. However, it is worth noting that this pattern does not apply to *P. kudriavzevii* EXF-9458, as this strain metabolizes 71% of butyric acid but only 40% of propionic acid. This suggests that the type of SCFA, whether it is even or odd-chain, also influences yeast metabolism^39^.

Increasing the SCFA concentration from 15 to 25 g/L did not result in higher biomass in the case of strains *Y. lipolytica* ZIM2116, *P. kudriavzevii* EXF-6398 and *P. kudriavzevii* EXF-6471. Moreover, their total consumption of SCFAs decreased from 60% in media with 15 g/L carbon source to 26% in media with 25 g/L SCFAs. However, with the exception of these three strains, all others exhibited an increase in the maximum OD_600_, with the *P. kudriavzevii* EXF-9458 strain particularly standing out. The latter strain produced a 4.7-fold more biomass at 25 g/L of SCFAs compared to 15 g/L. This could be due to a higher toxicity tolerance or indicate a faster uptake and metabolic processing.

**Figure 7:**
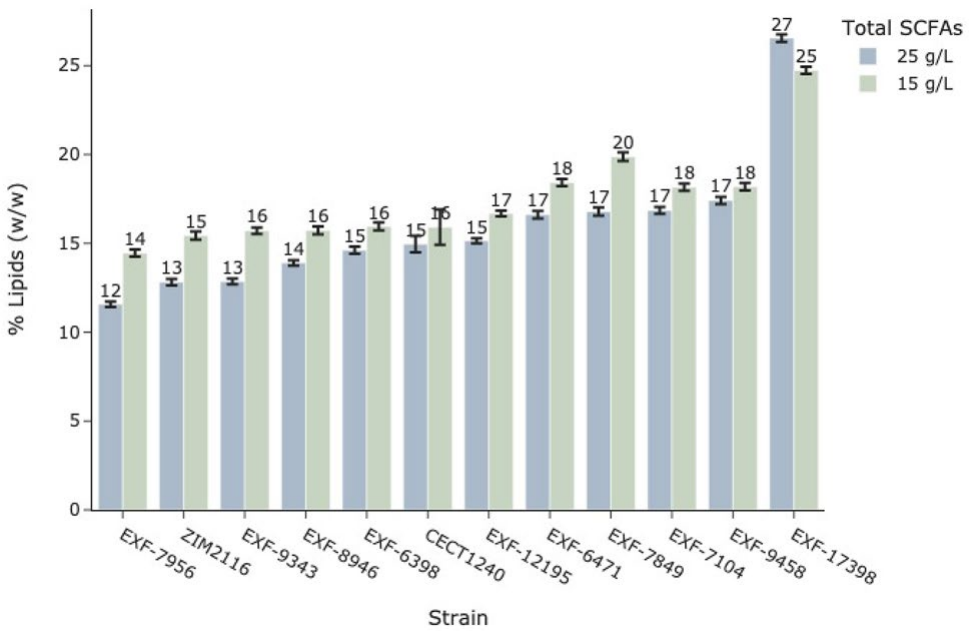
Lipid accumulation in yeast strains cultivated in 100 mL SCFA-rich media. Percentage of lipids (w/w) accumulated by most promising 11 yeast strains from the screening when grown in a synthetic medium with two total concentration thresholds of SCFAs, 25 g/L and 15 g/L, as the only carbon source. The carbon ratio between individual SCFAs: acetic, propionic and butyric acid was A:P:B = 3:1:1. Each bar represents the average lipid content with error bars indicating standard deviation.

On the other hand, the strain EXF-17398 achieved the highest intracellular lipid accumulation at 26.5%, which corresponds to an increase of almost 2% compared to the condition with 15 g/L SCFAs (Table 2 & 3, Fig. 7). However, no statistically significant differences were observed for the other strains.

### Improvement in lipid production through C/N ratio adjustment

It is well known that a higher C/N ratio increases lipid production in oleaginous yeasts^17,30,40–43^. Therefore, in order to increase lipid accumulation using SCFAs as the carbon source, the two best strains from the previous characterisation, *Y. lipolytica* EXF-17398 and *P. manshurica* EXF-7849, were grown in the medium containing 25 g/L SCFAs with a C/N ratio of 150:1 and 200:1 (Table 4), instead of the 5:1 ratio of the original screening medium described above.

**Table 4:** Consumption of SCFAs, maximal optical density (OD_600_), lipid content, yield and SCFAs consumption rate of the two most promising yeast strains, analysed at the end of cultivation in the screening media containing a total of 25 g/L SCFAs and acetic, propionic and butyric acid ratio of 3:1:1 with C/N adjustment. Y_l/s_:g lipid/g SCFAs.

Collection ID	Scientific name	C/N	Consumption of SCFAs (%)				Maximal OD600	Lipid content	Lipid yield	SCFAs consum. rate
								(%, w/w)	(Y_L/S_)	(g/L*h)
			Acetic acid	Propionic acid	Butyric acid	Total				
EXF-7849	*Pichia manshurica*	150	64	< 10	< 10	42	4.5 ± 0.8	11.0 ± 3.2	0.02	0.06
EXF-7849	*Pichia manshurica*	200	82	< 10	< 10	48	4.6 ± 0.2	21.0 ± 3.2	0.08	0.05
EXF-17398	*Yarrowia lipolytica*	150	100	42	62	80	13.7 ± 1.5	25.3 ± 3.8	0.04	0.09
EXF-17398	*Yarrowia lipolytica*	200	87	17	33	62	13.4 ± 0.3	29.0 ± 0.9	0.12	0.08

Increasing the C/N ratio to 150:1 had no significant effect on the lipid content of the yeast (*p* value > 0.05) compared to the medium without C/N adjustment (Fig. 7). However, when the C/N ratio was further increased to 200:1, lipid accumulation increased. In these conditions, *Y. lipolytica* EXF-17398 reached a lipid content of 29% w/w, compared to 25.3% w/w at 150:1. The lipid content of *P. manshurica* EXF-7849 increased from 11% w/w at 150:1 to 21% w/w at 200:1. Our two candidate strains therefore showed considerable lipid accumulation under stringent conditions with a high concentration of SCFAs at 25 g/L, including a high amount of butyric acid. The *Y. lipolytica* EXF-17398 strain reached a lipid content of 29% w/w under acidic conditions, which is comparable to other Y*. lipolytica strains* under more favourable alkaline conditions. The corresponding lipid yields for *Y. lipolytica* EXF-17398 and *P. manshurica* EXF-7849 were 0.12 and 0.08 g/g respectively. In comparison, the lipid yield using glucose can reach up to 0.32 g/g^44^, while the yield from acetic acid and mixed SCFAs under optimized conditions have been reported to be 0.14 and 0.16 g/g, respectively^23^. These values are comparable to the yield obtained in our study with *Y. lipolytica* EXF-17398.

The disadvantage of a higher C/N ratio is nitrogen limitation, which triggers lipogenic metabolic pathways, reduces biomass production and slows the growth rate of the yeast^3,19,20,36,45^. For this reason, the maximum OD_600_ of *Y. lipolytica* EXF-17398 decreased from 25.4 in the non-adjusted C/N to 13.4 in the C/N adjusted 200:1 medium. For *P. manshurica* EXF-7849, the reduction was from 10.5 to 4.6 under the same conditions.

The carbon sources remained partially unutilized in the medium with a C/N ratio of 200:1 (Table 4). *P. manshurica* EXF-7849 consumed 48% and *Y. lipolytica* EXF-17398 consumed 62% of carbon sources.

The fatty acid profiles of *P. manshurica* EXF-7849 and *Y. lipolytica* EXF-17398 revealed that both strains produced predominantly oleic acid (C18:1cis; 34.91% and 39.04%), linoleic acid (C18:2cis; 25.05% and 26.84%), and palmitic acid (C16:0; 11.95% and 11.55%) (Supplementary data S2, Table S2). These compositions are similar to those of vegetable oils, such as soybean and corn oil^22^. Notably, *P. manshurica* EXF-7849 also synthesized a considerable amount of linolenic acids, C18:3n3 (7.84%) and C18:3n6 (1.42%), which were not present in *Y. lipolytica* EXF-17398. It is also noteworthy that *Y. lipolytica* EXF-17398 produced odd-chain fatty acids, such as C17:0 and C17:1. This is likely due to its higher propionic acid consumption (Table 4)^46^. The different fatty acid compositions in yeasts are influenced by several factors, including pH, temperature, and C/N ratio. Studies have shown that adjustments to these parameters can alter the ratio of saturated to unsaturated fatty acids^22^. Further optimization of cultivation conditions in a bioreactor could therefore potentially improve the consumption of carbon sources and redirect it more efficiently towards lipid production.

Our research not only contributes to expanding the knowledge base on potential yeast species for microbial oil production from waste derived SCFAs, but also demonstrates an effective approach to obtain new biotechnologically promising yeast strains for competitive microbial oil production.

## Materials and methods

### Yeast strains used in this study

A total of 1434 natural yeast strains of various phylogenetic groups were included in this study. The criteria for selection were contingent upon their accessibility. 1,052 of them were obtained from the Culture Collection Ex (part of Infrastructural Centre Mycosmo, MRIC UL, Slovenia) and 382 strains from the Collection of Industrial Microorganisms ZIM (Slovenia). All tested strains with corresponding collection IDs and taxonomic lineage are listed in Supplementary data S1*. Y. lipolytica* strain CECT1240 from The Spanish Type Culture Collection was used as a reference strain given its ability to accumulate lipids above 20% w/w CDW and its capacity to efficiently utilize SCFAs as the only carbon source^36^. Yeast strains were arrayed to 384-well plates and kept in 30% (v/v) glycerol at − 80 °C until use, unless stated otherwise.

The taxonomic distribution of the tested strains is visualized using the *Metacoder* (v. 0.3.6)^45^ tool (Fig. 1, Supplementary data S2 Figure S1 and S2). It represents the number of strains within each taxonomic group, as indicated by the colour intensity and node sizes. The spatial arrangement of taxonomic groups within the figure does not imply phylogenetic relationships or distances.

### Growth assessment on short-chain fatty acids

Pre-inoculum cultures of each strain were obtained by cultivation on solid YPD (10 g/L yeast extract, 20 g/L peptone, 20 g/L glucose, 20 g/L agar) media at 30 °C until late exponential phase. Using a custom-made robotic hand with a 384 floating pins replicator (0.787 mm diameter pins, V&P Scientific), cells were transferred onto solid medium consisting of 6.9 g/L yeast nitrogen base (YNB) w/o amino acids, 7.5 g/L ammonium sulphate and 20 g/L agar. In addition to this, the control medium contained 20 g/L glucose, and the testing SCFA-rich media contained three individual SCFAs—acetic acid (C2), propionic acid (C3) and butyric acid (C4) at a final total concentration of 25 g/L and 15 g/L. Based on the carbon ratio between selected SCFAs, three different ratios of SCFAs were used: acetic (A) : propionic (P) : butyric (B) = 2:1:2, A:P:B = 3:1:1 and A:P:B = 1:1:3. The pH of the media was adjusted to 6.0 with 4 M NaOH.

For the initial growth assessment cells were pinned to a density of 1,536 colonies per plate and cultivated at 25 °C for 5 days in at least three biological replicates. Analysis was conducted in parallel for control (glucose) and testing media with 15 g/L and 25 g/L concentrations of SCFAs, and all three combinations of SCFA ratios as described above (6 SCFA-rich media in total). Subsequent screenings were then performed in three biological replicates with cells pinned to a density of either 384 or 96 colonies per plate and incubated at 30 °C for 5 days. Only the 25 g/L of SCFA-rich media was used in this latter case.

The plates with arrayed yeast colonies were scanned with the Epson Perfection V700 Photo scanner and analysed using the *pyphe* tool (v. 0.98) for high-throughput colony size growth quantification^35^. For the initial screening, plates were scanned and quantified once in batch mode after 5 days of cultivation. Colony sizes were computed where growth was observed with the *pyphe-quantify* function and parameters d = 2, s = 0.5. Yeast strains capable of growing on SCFAs after the initial screening were further analysed and scanned in a time-course mode every 15 min for 4 days. Growth parameters lag phase duration (lag), time at maximal growth (t_max) and fitness (colony size), were obtained with *pyphe-scan-timecourse*, *pyphe-growthcurves* and *pyphe-quantify* functions, respectively. Relative fitness was determined by normalising fitness values of strains on SCFA-rich media composition to their fitness on glucose. Fold changes of growth parameters were computed by division and subtraction of 1, for comparison between candidate strains and the reference strain.

For the 571 yeast strains with inconsistent growth after the initial screening, manual application to solid SCFA-rich media composition A:P:B = 1:1:3 at a final total concentration of 25 g/L SCFAs was performed. Of these 571 strains, all 35 *Yarrowia* strains were subjected to manual application to all three SCFA-rich media compositions listed above at the total 25 g/L SCFAs concentration.

### Lipid accumulation assay

The accumulation of neutral lipids was determined for the 54 strains with best growth characteristics on the SCFA-rich media and the reference yeast strains capable of growing on SCFAs after the initial screening. For this, a semi-high-throughput approach, based on the Nile Red (NR) assay was used as described by Pačnik et al.^47^. Briefly, yeast cells were cultivated in minimal medium (MM-P) with 6.9 g/L YNB w/o AA (with 5 g/L ammonium sulphate), 10 mg/L myo-inositol, 20 g/L glucose and 2 g/L amino acids mixture^47^. MM-P was buffered to pH = 5.7 with 20 mM 2-(N-morpholino) ethane sulfonic acid (MES) buffer. Cells were cultivated in 96-well microtiter plates (V = 0.25 mL) covered with a gas permeable sealing membrane and cultivated at 30 °C and 180 rpm agitation for 72 h. Cell cultures were then transferred to black clear-bottom 96-well microtiter plates and diluted to optical density 595 nm (OD_595_) 0.6 ± 0.2 with dimethyl sulfoxide (DMSO) to a final concentration of 20% (v/v).

After background fluorescence measurements, Nile red dye was added to a final concentration of 5 μg/mL (2.1 mg/mL stock solution, dissolved in 99.5% DMSO) and plates were incubated for 25 min in the dark. Tecan Infinite^®^ M1000 microplate reader was used for fluorescence measurements at excitation λ = 485 ± 10 nm and emission λ = 535 ± 10 nm. Absorbance was measured as OD_595_. Relative Fluorescence Units (RFU) were calculated by subtracting the background fluorescence and normalising to biomass OD_595_ values.

### Fermentation media and conditions

Fermentation experiments were performed using 100 mL SCFAs-rich synthetic media in 250-mL Erlenmeyer flasks with baffles to promote aeration. The SCFAs-rich synthetic media was composed of 17 g/L YNB w/o AA solution with 15 g/L or 25 g/L SCFAs containing acetic, propionic and butyric acid at a 3:1:1 ratio. The concentration of added ammonium sulphate was adjusted to set a C:N ratio of 150:1 or 200:1.

For all fermentation tests, initial pH was adjusted to 6.0 with 3 M NaOH. During the fermentation process the pH was not monitored. Fermentation was initiated by inoculating yeast cells from pre-inoculum whereby cells were diluted to an optical density of 1 at 600 nm (OD_600_) (corresponding to 0.45 g DW cells/L). Yeast growth was evaluated by measuring OD_600_ of the cultures (Spectroquant^®^ Pharo 100 spectrophotometer). Experiments were carried out in a rotary shaker at 170 rpm and 27 °C until 60—70% of the carbon source was consumed (monitored as described below).

### Short-chain fatty acid determination

SCFAs were determined by liquid chromatography with an Agilent 1260 HPLC-RID (Agilent, Santa Clara, CA, USA) equipped with a Cation H Refill Cartridge Microguard column (Bio-rad, Hercules, CA, USA) and an Aminex HPX-87H ion exclusion column (300 × 7.8 mm I.D.) (Bio-rad). The mobile phase was 5 mM H_2_SO_4_ and elution was performed in isocratic mode at a flow rate of 0.6 mL/min. The injected sample volume was 20 μL. The oven and detector were set at 44 °C and 35 °C, respectively.

### Lipid extraction and gravimetric determination

Due to the lack of standard curves for the lipid determination by fluorescence for all the tested strains, lipid content was determined gravimetrically as detailed in Morales-Palomo et al.^48^. Briefly, cell biomass (0.4 g/L) was centrifuged at 5000 rpm for 10 min at 4 °C (Heraeus, Megafuge, Thermo Scientific) and dried at 105 °C overnight. Dry biomass (around 0.2 g dry weight) was mixed with a solution of chloroform and methanol (2:1 v/v) under reflux for 4 h. The extract was filtered (Watman N.1 paper) and 0.9% NaCl solution was used to wash the organic phase. Samples were dried using anhydrous Na_2_SO_4_ (Sigma). The chloroform phase containing the lipids was evaporated using a Rotavapor R-215 (BUCHI) at 40 °C and 350 mb of vacuum. Finally, total cellular lipids were gravimetrically determined and expressed as grams of lipid per grams of dry biomass (% w/w).

### Fatty acid determination

The fatty acid composition in the lipids was determined by gas chromatography (GC) after fatty acid *trans*-methylation. For this purpose, a pellet of the biomass sample was kept at – 80 °C for 24 h. Then the cells were lyophilized, grounded and 10–20 mg of ground cells were used for the analysis. Tridecanoic acid (C13) was used as internal standard. To each sample, 15 μL C13, 400 μL chloroform:methanol (2:1) and 600 μL HCl:chloroform were added. The mixture was gently mixed and digested for 1 h at 90 °C. The mixture was cooled before the adding 2 mL of hexane. After gently mixing, the mixture was allowed to stand for more than 1 h to allow separation of the two phases. An aliquot of the upper layer was removed and transferred to a GC vial.

The samples were analysed in an Agilent 7890 AGC system equipped with a J&W column (30 m × 0,25 mm d × 0.25 µm DB-23 part: 122–2332) and an FID detector. The GC conditions were as follows: Helium was used as the carrier gas at a constant pressure (33.359 psi), the FID temperature was 280 °C and the oven temperature was 230 °C. The peaks of the FA methyl esters were identified by comparison with the standards.

### Statistical analyses

Median growth parameter values as well as lipid accumulation RFU values were obtained and then scaled using the MaxAbsScaler function from the scikit-learn package (v.1.2.0) for comparison. The values were also compared to the values of the reference strain CECT1240.

The normality Shapiro–Wilk test and variance homogeneity Levene’s test were performed at a significance level 0.05 for scaled phenotypic data from the large-scale screening analysis. Then a non-parametric Mann–Whitney U test was performed to analyse differences between candidate yeast strains and the reference CECT1240 strain. Significance threshold was again set at 0.05. Differences were considered significant if Benjamini–Hochberg corrected *p* value was smaller than 0.05.

A parametric one-way ANOVA, F-test and Kruskal–Wallis test were used for the assessment of means and variances of microbial lipid content (% w/w) (confidence interval 90%), respectively. Differences were considered significant at *p* value < 0.05.

### Weighted ranking

Candidate yeast strains were ranked in relation to two traits—effective utilisation of SCFAs and quantity of accumulated neutral lipids. Weighted ranking was performed by assigning lipid accumulation a greater importance. For this, fitness (colony size) and time at maximal growth (t_max), calculated by *pyphe* as described above, on all three media (with different SCFA compositions at 25 g/L), as well as lipid content (expressed as RFU), were chosen as variables. First, the candidate strains were sorted based on the selected variables. Lipid content and fitness values were sorted in descending order, while t_max values were sorted in ascending order. Sorted yeast strains were then ranked in descending order for each of the variables of the corresponding SCFA-rich media composition separately. Importance weights were then applied by multiplying generated ranks of fitness and t_max variables with k = 1.2 (40% total importance; i.e., 20% importance each) and lipid content variable with k = 1.6 (60% importance). Finally, weighted variable ranks were summarised for each strain and ranked again in descending order with a higher rank indicating better overall performance. The top 11 strains were chosen for further fermentation experiments.

### Supplementary Information

Supplementary Information 1.

Supplementary Information 2.

## Data Availability

All the data reported in this study are included in the article and its supplementary information files. The complete database with all taxonomic and phenotypic data as well as statistical data has been provided in Supplementary File S1. Additional supplementary data supporting the findings of this study are available in Supplementary Data S2. Both files were deposited to Open Science Foundation (OSF) repository and are accessible for review at 10.17605/OSF.IO/FXTR5.
