# Supplementary material for: High-throughput screening of non-conventional yeasts for conversion of organic waste to microbial oils via carboxylate platform
Source: Sci Rep. 2024 Jun 20;14:14233. doi: 10.1038/s41598-024-65150-w (PMC11190255; doi:10.1038/s41598-024-65150-w)
Supplement: Supplementary file 2 — Supplementary Information 2. [file 41598_2024_65150_MOESM2_ESM.docx]

## High-throughput screening of non-conventional yeasts for the conversion of organic waste to microbial oils via carboxylate platform

Mia Žganjar^1,2^, Mojca Ogrizović^1^, Mojca Matul^3^, Neža Čadež^2^, Nina Gunde-Cimerman^3^, Cristina González-Fernández^4,5^, Cene Gostinčar^3^, Elia Tomás-Pejó^4^, Uroš Petrovič^1,3,*^

^1^ Jožef Stefan Institute, Department of Molecular and Biomedical Sciences, Slovenia

^2^ University of Ljubljana, Biotechnical Faculty, Department of Food Science and Technology, Slovenia

^3^ University of Ljubljana, Biotechnical Faculty, Department of Biology, Slovenia

^4^ IMDEA Energy, Biotechnological Processes Unit, Spain

^5^ Institute of Sustainable Processes, Dr. Mergelina, Spain

* Corresponding author e-mail address: [uros.petrovic@bf.uni-lj.si](mailto:uros.petrovic@bf.uni-lj.si)

Supplementary information S2

^
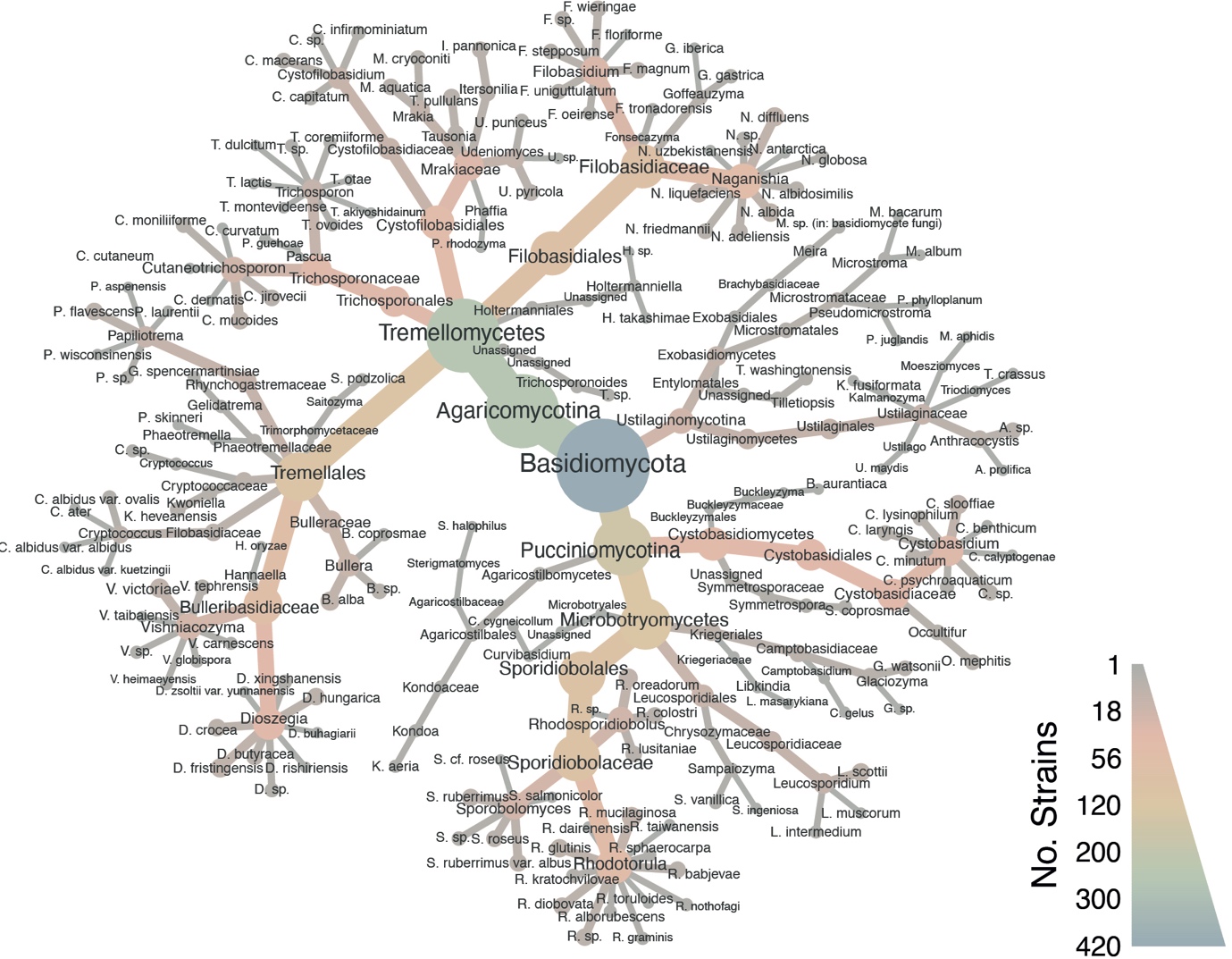
^

**Figure S1** **The taxonomic distribution of tested yeast strains of Phylum Basidiomycota.** Colour scale and node size denote number of strains per taxa. Spatial arrangement of taxonomic groups does not imply phylogenetic distances.


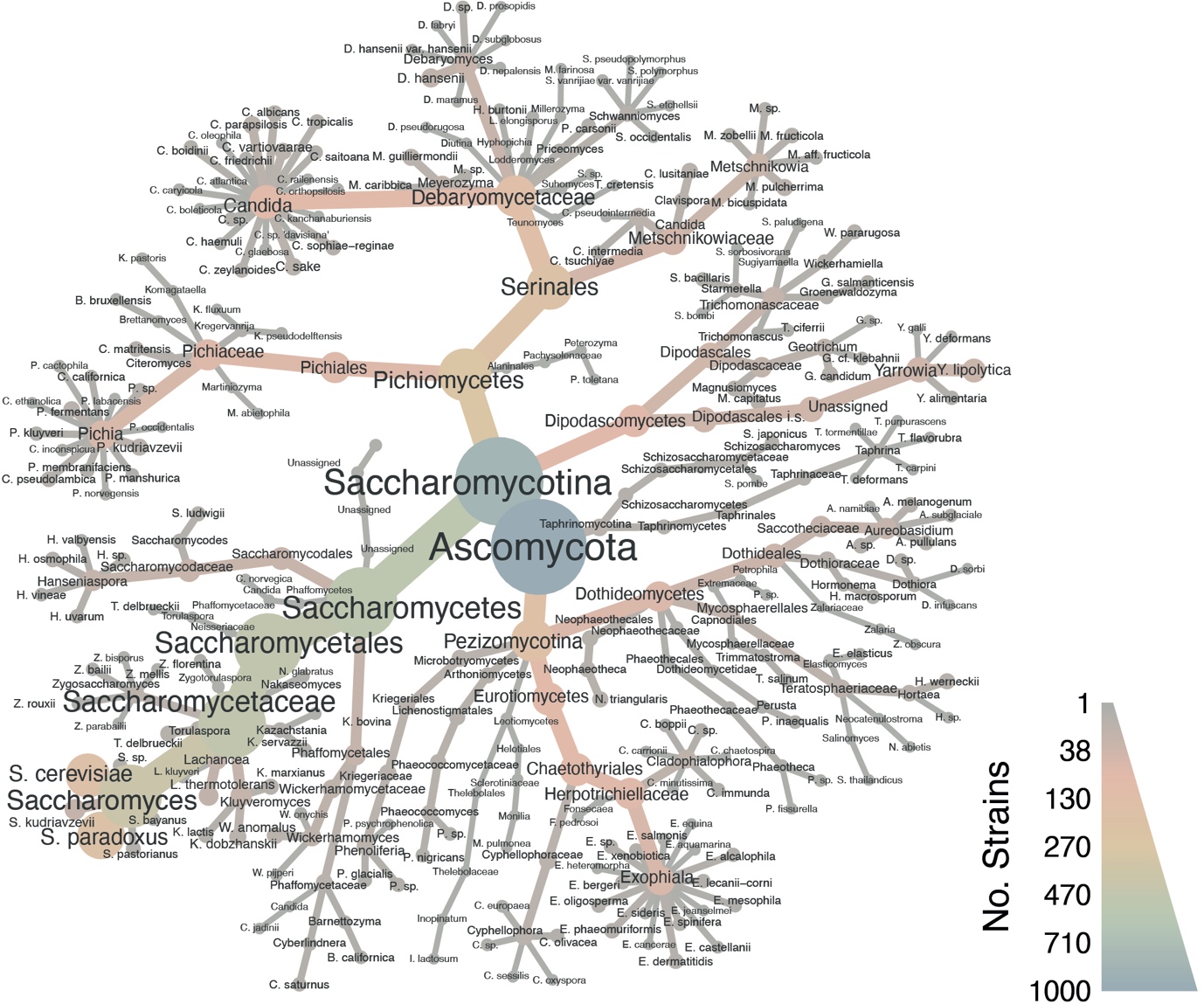


**Figure S2** **The taxonomic distribution of tested yeast strains of Phylum Ascomycota.** Colour scale and node size denote number of strains per taxa. Spatial arrangement of taxonomic groups does not imply phylogenetic distances.


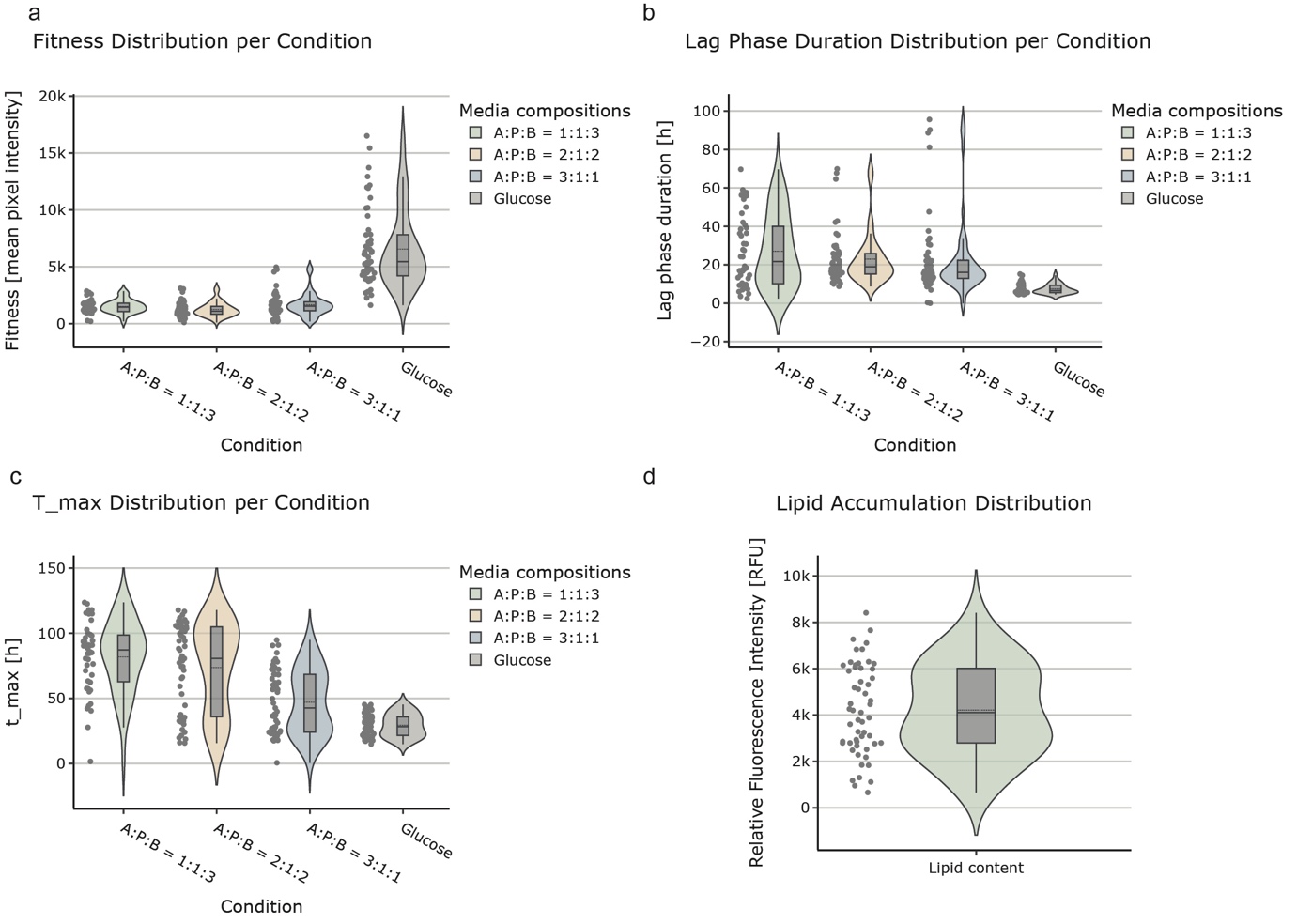


**Figure S3 Phenotype data distributions of 54 candidate yeast strains with Y. lipolytica CECT1240 as a reference strain.** Violin plots represent phenotype variability per media composition for three main growth parameters: a) fitness, b) time at maximal growth (t_max) and c) duration of lag phase (lag). Additionally, d) distribution of lipid accumulation is presented. Tested media compositions represent the SCFA-rich synthetic media with acetic (A), propionic (P) and butyric (B) acids ratios of: A:P:B =3:1:1, A:P:B =2:1:2, A:P:B =1:1:3. Control condition contains glucose as sole carbon source.

***
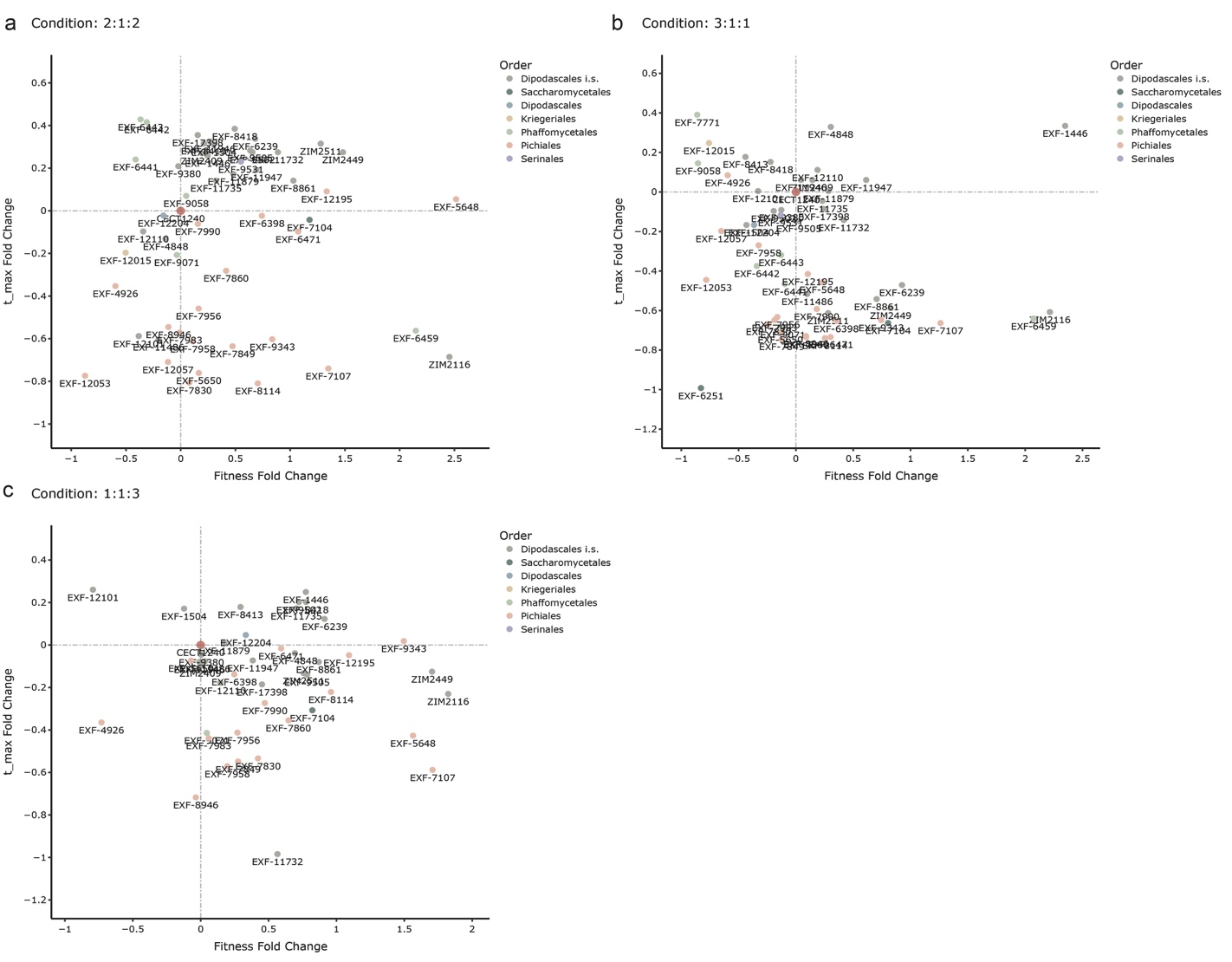
***

**Figure S4 Relationship between fitness and time at maximal growth (t_max) compared to the Y. lipolytica CECT1240 reference strain per SCFA-rich media composition.** Fold Change values of fitness and T-max values normalised to reference strain, denoted with a red dot, are presented. Strains are coloured according to their classification to Order.

**Table S1 Identity and number of natural yeast strains selected after the initial large-scale screening process with the reference strain Y. lipolytica CECT1240 included.**

| **Genus** | **Species** | **No. of strains** |
| --- | --- | --- |
| ***Candida*** | *orthopsilosis* | 1 |
| ***Nakaseomyces*** | *glabratus* | 2 |
| ***Phenoliferia*** | *glacialis* | 1 |
| ***Pichia*** | *cactophila* | 1 |
|  | *californica* | 2 |
|  | *ethanolica* | 1 |
|  | *inconspicua* | 1 |
|  | *kudriavzevii* | 4 |
|  | *manshurica* | 3 |
|  | *membranifaciens* | 1 |
|  | *norvegensis* | 1 |
|  | *occidentalis* | 1 |
|  | *pseudolambica* | 4 |
|  | **Pichia Total** | 19 |
| ***Wickerhamiella*** | *pararugosa* | 1 |
| ***Wickerhamomyces*** | *anomalus* | 7 |
| ***Yarrowia*** | *deformans* | 1 |
|  | *galli* | 1 |
|  | *lipolytica* | 22 |
|  | **Yarrowia Total** | 24 |
|  | **Grand Total** | 55 |

**Table S2 The fatty acid composition of lipids produced by Y. lipolytica EXF-17398 and P. manshurica EXF-7849 from medium containing a total of 25 g/L SCFAs and A:P:B = 3:1:1.**

|  |  | **Relative amount of fatty acids (% w/w)** | | | | | | | | | | | | |
| --- | --- | --- | --- | --- | --- | --- | --- | --- | --- | --- | --- | --- | --- | --- |
| **Species** | **Strain ID** | **C14:0** | **C15:0** | **C16:0** | **C16:1** | **C17:0** | **C17:1** | **C18:0** | **C18:1cis** | **C18:2trans** | **C18:2cis** | **C18:3n6** | **C18:3n3** | **C20:1n9** |
| *Y. lipolytica* | EXF-17398 | 1,94±0.05 | 1.45±0.03 | 11.55±0.05 | 8.36±0.02 | 1.13±0.03 | 2.45±0.01 | 3.19±0.05 | 39.04±0.11 | 0.00 | 26.84±0.16 | 0.00 | 0.00 | 1.15±0.03 |
| *P. manshurica* | EXF-7849 | 2,07±0.06 | 0.00 | 11.95±0.03 | 9.88±0.04 | 0.00 | 0.00 | 5.69±0.06 | 34.91±0.05 | 1.21±0.02 | 25.05±0.14 | 1.42±0.09 | 7.84±0.03 | 0.00 |
